# Uncomfortable Truths about Modern Epidemics: A Review of *The Pandemic Century* and Interview with Author Mark Honigsbaum

**DOI:** 10.4269/ajtmh.19-0388

**Published:** 2019-07-22

**Authors:** Claire Panosian Dunavan

**Affiliations:** University of California; Los Angeles, California; E-mail: cpanosian@mednet.ucla.edu

In December 2017, then-ASTMH President Regina Rabinovich calmly read the tea leaves for the coming year. Her first observation—“There will be epidemics…”— became a tag-line for our 2018 meeting. In the meantime, Gina’s unblinking forecast made me wonder just a little harder about the next blight to strike. And the next. The exercise was both useful and disturbing, because it forced me to ponder not only microbes but modern life.

Reading Mark Honigsbaum’s latest book, one learns that looking back at epidemics can be equally useful and disturbing. In *The Pandemic Century: One Hundred Years of Panic, Hysteria, and Hubris* (W.W. Norton, 2019), the journalist and author-turned-infectious diseases historian not only revisits important global outbreaks but interrogates their social and cultural catalysts and scientific blind spots. The result is an important, riveting, and deeply-researched work that will likely be read for years to come.

Now for my disclosures. As a former history major, I believe that medical scientists in general and tropical medicine specialists in particular ignore history at their peril. Plus I am already Honigsbaum’s fan, having praised his first book—*The Fever Trail: In Search of the Cure for Malaria* (Farrar, Straus and Giroux, 2002)*–*in the Los Angeles Times and Scientific American. *The Fever Trail* chronicled the high-stakes, 19^th^ century search for *Cinchona ledgeriana,* the elusive tree whose bark yields quinine. Criss-crossing South American jungles and cloud forest, three rivals led the race: Richard Spruce, a hypochondriacal moss collector; Charles Ledger, a cockney fortune-hunter; and Clements Markham, a high-minded geographer. Honigsbaum’s colorful narrative kept me glued to the page as I followed the men and the life-saving alkaloid eventually extracted after cinchona seedlings were transplanted to Dutch plantations on Java. Today, I would still commend *The Fever Trail* to anyone remotely involved with malaria.

*The Pandemic Century*, in contrast, is epic in scope, spanning Spanish flu, parrot fever, and plague to Legionnaires Disease and SARS to HIV/AIDS, Ebola, and Zika. Whereas *The Fever Trail* is a natural history thriller with noir-ish undernotes of commerce and greed, what distinguishes Honigsbaum’s new work is its dogged search for truths that some may find edgy. To what extent do past experiences and faith in technological tools *du jour* blind us to new ecologies, behaviors, and pathogens still waiting to burst on the stage? the author repeatedly asks, responding with a feast of information from earlier medical infernos. For serious students of the field, this is epidemiology at its richest.

Standing back, one could also liken Honigsbaum’s quest with current efforts to gauge how income, early childhood development, education, employment, housing, gender, and race impact 21^st^ century health. At the same time conceding that “…’nature’ is the greatest bioterrorist of them all,”^1^ Honigsbaum shows how other byproducts of contemporary life—think roads and transportation; urbanization and crowding; healthcare facilities; religious and ethnic tensions; media; climate change, and the list goes on—have helped to fuel modern pandemics.

Of course, if we’re going to think big about social determinants of pandemics, why not think really big? One of the uber-morals of *The Fever Trail* is how people and organizations often seek selfish gain over collective good. Remember Charles Markham, the third man in pursuit of the life-giving tree with the cinnamon bark and crimson-lined leaves? His vision (shared by Florence Nightingale, no less) was to right the wrongs inflicted on Britain’s colonial subjects by providing them with better sanitation and low-cost malaria treatment. Sadly, years later, his dream was dashed. Although the worldwide price of quinine had indeed plummeted thanks to Java’s prolific groves of *C. ledgeriana*, malaria’s toll had not. In Honigsbaum’s words: “There was a limit to philanthropy and … the British had reached it. If any profits were to be made from the bark in the future, they would come from selling quinine to those who could afford it—in other words, ‘rich’ Europeans and Americans.”

The ecologies of human infectious diseases—both those that are stubbornly entrenched and those that go rogue—are multi-layered indeed.

## SIX QUESTIONS FOR MARK HONIGSBAUM

**Please describe your dual career. What sparked your interest in the history of medicine and infectious diseases, and what are your most essential tools?** My father was a historian of medicine so you could say it was in my DNA.^2^ But after finishing university, the last thing I wanted was to spend any more time in a library so I opted for journalism, and except for a chance encounter with a botanist while on assignment in Switzerland, I may never have followed in my father’s footsteps. As I wrote in *The Fever Trail*, while sharing a table in a Zurich pizzeria, the botanist regaled me with the story of cinchona, the tree which, for centuries, was the only cure for malaria.

Long story short, on my return to London I visited the Wellcome Library and discovered that long before Henry Wellcome had traveled to South America in the 1870s in search of cinchona, there had been other expeditions to the Andes. I learned more at the Royal Geographical Society and at Kew Gardens, but the best resource was the London School of Hygiene and Tropical Medicine. Before traveling to the Amazon and the Andes, I spent weeks there perusing old books on malaria and interviewing malariologists.

What really solidified my interest [in infectious diseases], however, were the 2005 bird flu outbreaks. By then I was back at the Guardian working as a journalist. Those outbreaks – and the hysterical reporting that accompanied them – convinced me to pursue a PhD on the history of influenza. My doctoral work was my true apprenticeship. Not only was I introduced to the founding fathers of the history of medicine, I began reading medical anthropology and the social and cultural history of medicine. My deepest insights came from medical sociology and science and technology studies, in particular, Ludwick Fleck’s *Genesis and Development of a Scientific Fact.* I now regard these as my essential tools.

**What were your goals in writing *The Fever Trail* compared with *The Pandemic Century.*** In *The Fever Trail,* I wanted to tell a little-known story with huge military, economic, and political consequences, particularly in India and Southeast Asia, where malaria impeded the efficiency of colonial administrations. It was essentially a narrative-led history.

By contrast, *The Pandemic Century* reconstructs epidemics and pandemics from the ground up while attempting to avoid the tropes of the ‘outbreak narrative.’ Drawing on studies of science and technology and the theory of knowledge, I tried to show how in each of the epidemics and pandemics, with the exception of HIV/AIDS, medical researchers were blinded by scientific paradigms and limited laboratory tools. I also wanted to challenge the current global health security discourse by reminding readers of the insights of mid-20^th^ century, ecologically-minded researchers such as Rene Dubos, Karl Friedrick Meyer, and Frank MacFarlane Burnet. So, while this is a book for the general reader, I hope it will also reverberate with medical scientists, medical historians, and students of global health.

**Was there a single ‘aha’ moment?** Not really; it was more an accretion of ‘ahas’ because, for some time, I had been interested in the relationship between science and the media with respect to messaging risk. In the 1889-92 Russian influenza pandemic, telegraphic communications helped provoke a popular dread of the flu – the Lancet went as far as to call the Russian flu “an epidemic started by telegraph.” However, it was the 2014 Ebola epidemic that crystalized my ideas about the role of scientific knowledge in generating complacency as well as anxiety about epidemic threats. The key moment came in May 2014 when I attended a meeting on Ebola at Chatham House and all the experts essentially said of the outbreak in southeastern Guinea, “we’ve seen this before in other parts of Africa; there’s nothing to worry about.”

Three months later Ebola arrived in Monrovia and Freetown and everyone started blaming the WHO for failing to appreciate the danger. But I firmly believe the fault had less to do with the WHO than with the scientific construction of Ebola as a rare hemorrhagic virus and the way we are continually being led astray by our experience of previous epidemics.

**Let’s discuss the many trips you took before writing this book along with the interviews you conducted and the materials you reviewed.** The book combines deep archival research with reportage and interviews with scientists and health experts. Although I had already written two books on influenza, for the first chapter on the Spanish flu I found myself revisiting surveys and reports by medical officers attached to the US Army and the Surgeon General’s Office in order to tell the story from an epidemiological and immunological point of view; at the same time I interviewed contemporary flu experts for the latest epidemiological and virological insights. For the chapters on the 1924 epidemic of plague in Los Angeles and the 1930 parrot fever pandemic, I drew on papers at the Bancroft Library in San Francisco and the Huntington Library in Pasadena. At the Smithsonian Library I found several boxes of CDC records documenting its investigation of the 1976 Legionnaire’s Disease outbreak in Philadelphia and I also interviewed David Fraser, who headed the investigation, and Joe McDade, the microbiologist who eventually solved the puzzle. In the case of the Ebola chapter, I was fortunate to have visited Sierra Leone in 2015 in order to conduct interviews for an oral history project for the Wellcome Trust and had also traveled to the outbreak’s epicenter in Kenema. For the SARS chapter, I visited Hong Kong; for the Zika chapter, I visited Recife, Brazil.

Although I could have reconstructed these outbreaks from my desk in London, there is no substitute for walking the streets and talking to the scientists who were there at the time: the epidemics I canvass are as much about people, place, and the built environment, as the ecology of the pathogens. In addition, it’s only by visiting an outbreak zone that you begin to understand the environmental and social conditions that fuel these epidemics and their impacts on local clinicians and responders.

**How can we train future generations to better anticipate pandemics and their proximate causes? How can we better inform the public about their inevitability?** A lot of this work is already being done by organizations like the Coalition for Epidemic Preparedness Innovation (CEPI) and Prevent. And the WHO has got a lot better at integrating medical anthropologists and social scientists into its epidemic response teams. But too many scientists still suffer from the delusion that they’re the good guys and it's simply a matter of the “natives” trusting their superior expertise. The world isn't like that any more. There is huge distrust of all forms of expertise and not just in Africa and Asia – you only have to look at the outbreaks of measles in the US and Europe due to people, many of them well-educated, refusing to vaccinate their children. High-quality journalism and better science reporting to combat online misinformation can make a difference, to be sure. But medical history and sociology also have a role to play, if only to remind scientists not to be too complacent about the progress we’ve made.

**If you could choose between sequencing a slew of viruses that could potentially cause our next pandemic and investing in a universal flu vaccine, which would you choose?** Flu, without a doubt. Influenza is a truly universal disease. No virus poses a greater threat to more people. It is only good fortune that we haven't seen another pandemic as severe as 1918.

## Footnotes

1 Peiris JSM, Guan Y. Confronting SARS: a view from Hong Kong, Philosophical Transactions of the Royal Society of Biology, 2004.

2 Frank Honigsbaum, *The Division in British Medicine : The Separation of General Practice from Hospital Care, 1911-1968* (London: Kogan Page, 1979) and *Health, Happiness And Security: the creation of the National Health Service* (London; New York: Routledge, 1989).

